# Eye movement corpora in Adyghe and Russian: an eye-tracking study of sentence reading in bilinguals

**DOI:** 10.3389/fpsyg.2023.1212701

**Published:** 2023-09-13

**Authors:** Nina Zdorova, Olga Parshina, Bela Ogly, Irina Bagirokova, Ekaterina Krasikova, Anastasiia Ziubanova, Shamset Unarokova, Susanna Makerova, Olga Dragoy

**Affiliations:** ^1^Center for Language and Brain, HSE University, Moscow, Russia; ^2^Institute of Linguistics, Russian Academy of Sciences, Moscow, Russia; ^3^Department of Psychology, Middlebury College, Middlebury, VT, United States; ^4^School of Linguistics, HSE University, Moscow, Russia; ^5^Laboratory of Experimental Linguistics, Adyghe State University, Maykop, Russia

**Keywords:** eye movement benchmarks, cross-linguistic study, universal patterns of reading, minority language, polysynthetic language, West Circassian

## Abstract

The present study expands the eye-tracking-while reading research toward less studied languages of different typological classes (polysynthetic Adyghe vs. synthetic Russian) that use a Cyrillic script. In the corpus reading data from the two languages, we confirmed the widely studied effects of word frequency and word length on eye movements in Adyghe-Russian bilingual individuals for both languages. We also confirmed morphological effects in Adyghe reading (part-of-speech class and the number of lexical affixes) that were previously shown in some morphologically-rich languages. Importantly, we demonstrated that bilinguals’ reading in Adyghe does differ quantitatively (the effect of language on reading times) and qualitatively (different effects of landing and previous/upcoming words on the eye movements within a current word) from their reading in Russian.

## Introduction

Recent eye-tracking studies have been specifically investigating universal patterns of reading across languages in monolingual (English in Cop et al., 2017; 13 languages in Siegelman et al., 2022) and bilingual individuals (Dutch-English in Cop et al., 2015; Chinese-English in Sui et al., 2022) within a corpus-based approach and traditional experimental paradigm [see a comparative study of reading in English, Finnish, and Chinese by Liversedge et al., 2016]. Whereas previous research has been done on major languages of language families, and on bilingual pairs using contrasted orthographies and morphological structures, the present study expands the eye-tracking-while-reading research toward less studied languages of different typological classes (polysynthetic Adyghe vs. synthetic Russian) with the same (Cyrillic) script.

Decades of eye-tracking research have already established psycholinguistic features that affect readers’ eye movements and, consequently, their language processing. The most robust lexical effects on eye-movements (i.e., the ones shown consistently across a range of empirical studies) are imposed by word frequency, word length and word predictability (Inhoff and Rayner, 1986; Rayner, 1998; Staub and Rayner, 2007). They were shown to affect both fixation durations and probabilities of skipping, i.e., the probability of a word being skipped and not fixated during the first-pass reading. Frequent words (Schilling et al., 1998), shorter words (Inhoff and Radach, 1998), and contextually more predictable words (Rayner and Well, 1996) are skipped more often and fixated for a shorter time (Clifton et al., 2007).

Apart from lexical features, morphological and morphosyntactic ones also affect eye movements across languages. For instance, verbs were shown to be read significantly slower than nouns in both Russian-speaking adults (Laurinavichyute et al., 2019) and children (Lopukhina et al., 2022). The latter also showed a difference in skipping rate based on the part-of-speech (POS) with verbs being less likely to be skipped than nouns (Lopukhina et al., 2022). Comparing two bigger groups of word classes (content words vs. function words) in English, Schmauder et al. (2000) reported that function words had a longer total reading time and were reread more frequently than content words. The study also found no evidence for a higher skipping rate on function words when frequency and length were controlled for.

To embrace a holistic approach to language reading with a range of linguistic features taken into account, reading corpora, also known as corpora of eye movements, have become a productive tool in the last decade [see The Multilingual Eye-tracking Corpus of eye movements while reading texts (MECO, Siegelman et al., 2022); Potsdam Sentence Corpus (Kliegl et al., 2004); Ghent corpus of bilingual text reading (Sui et al., 2022); Russian Sentence Corpus (RSC, Laurinavichyute et al., 2019); The child version of the Russian Sentence Corpus (ChiRSC, Lopukhina et al., 2022); The Bilingual Russian Sentence Corpus (BiRSC, Parshina et al., 2021) etc.]. Importantly, the corpora enable us to establish the basic characteristics of eye movements (eye movement benchmarks) and compare them across languages.

Crucially, disregarding the core idea of universality and language specificity that imply linguistic diversity as a necessary prerequisite, the languages in eye-tracking studies (incl. Eye-tracking corpora studies) are, so far, mostly Indo-European languages and the biggest representatives of Uralic, Sino-Tibetian, and Turkic language families, like Finnish, Chinese, Turkish etc. Moreover, the emphasis of the cross-linguistic comparison is primarily based on the differences in orthographies and scripts (English, Chinese, and Finnish in Liversedge et al., 2016; English and Russian in Parshina et al., 2021). Hence, a diversity in reading corpora, a focus shift on morphologically-driven cross-linguistic comparison of eye movements, and a greater attention to smaller representatives of language families, like minority languages is proposed.

The present study covers the eye movement benchmarks while reading in a polysynthetic minority language, Adyghe (also known as Adyghe),^1^ which has not been done before. Adyghe is one of the West Caucasian languages spoken in Russia and some Middle East countries. It is an SOV language spoken predominantly in southern Russia, by 81,294 people with 75,793 people using it on an everyday basis (according to the Russian Population Census, 2020).^2^ Adyghe uses the Cyrillic script, but includes some language-specific letters, and its orthography is opaque – i.e., the letter-phoneme correspondence is inconsistent and not transparent (Daniel and Lander, 2011; Polinsky, 2020). Adyghe includes the Bzhedugh, Shapsugh, Abadzekh, and Temirgoy dialects (Polinsky, 2020), where the latter is considered the standard variety.

As all Adyghe speakers are also Russian speakers (Polinsky, 2020), their reading data in both languages were collected and compared in within-language and within-group analyses. Russian is a Slavic synthetic SVO language with some analytic trends. It is based on a Cyrillic script, and its phoneme-letter correspondence allocates it to the language with medium-shallow orthography (Rakhlin et al., 2017; Zhukova and Grigorenko, 2019). The last years have seen a major growth in psycholinguistic studies of Russian, including three corpus studies of eye movements in different Russian-speaking populations (Laurinavichyute et al., 2019; Parshina et al., 2021; Lopukhina et al., 2022), that established eye movements benchmarks in Russian (summarized in Table 1) and described the contribution of lexical and morphosyntactic features into reading in Russian.

**Table 1:** Descriptive statistics of language use according to the shortened LEAP-Q form.

	Adyghe	Russian
Age of reading acquisition onset, years, Mean (SD)	7.2 (2.6)	5.9 (0.9)
Reading skill score, on scale 1 to 5 with 5 as the highest, Mean (SD)	4.0 (0.8)	4.8 (0.5)
Language use per day, %	58.6	41.4
Reading exposure per day, % of participants		
Almost none	4	0
<1 h	64	4
1–2 h	22	16
2–3 h	6	26
3–4 h	2	36
>4 h	2	18
Preferred language to read a text for pleasure, % of participants	16	84

To sum up, while there is some knowledge about the lexical, morphological, and morphosyntactic effects on eye movements during reading in different populations, languages, and orthographies, little is still known about the reading behavior of bilinguals in understudied, typologically different languages that use the same script. The goals of the study were, therefore, twofold. First, we aimed to establish benchmarks in eye movements while bilinguals were reading in a polysynthetic language (Adyghe) and report the psycholinguistic features that affect eye movements while reading in it. Second, we aimed to explore the differences between reading in two morphologically different languages (polysynthetic Adyghe vs. synthetic Russian), which are both Cyrillic-based.

A within-group comparison of reading bilinguals’ data in two languages enabled us to disentangle the effect of language, *per se*, and to shift from a common comparison of bilinguals with monolingual controls (Rothman et al., 2022). However, the discussion of the findings does rely on a meta-comparison with other Russian-speaking groups, like monolinguals (Laurinavichyute et al., 2019), Russian heritage speakers (HSs), and L2 learners of Russian (Parshina et al., 2021).

## Materials and methods

### Participants

Sixty five bilingual adult speakers of Russian and Adyghe took part in the study (57 women; Mean age = 30.2, SD = 13.5, range 18–60). The mean education level among participants was 14.9 years, SD = 2.3, range 11–20. All participants were recruited in Maykop, the capital of the Republic of Adygea, and they were primarily students of the Adygea State University (*N* = 32). The recruitment unfolded in 2 years: as a first stage of the study in 2021 and the final stage of data collection in 2022.

Whereas the majority indicated both Adyghe and Russian as their mother languages, 23 participants considered Adyghe as their only mother tongue, with Russian as their second language. At the same time, most participants’ family languages (i.e., languages spoken by their parents) were, again, both Adyghe and Russian (*N* = 57).

Fourteen participants indicated speaking more than one Adyghe dialect. In this case, we asked them to specify the one mostly used, rather preferred, and/or spoken in the family, which we considered as the dominant dialect. Hence, the distribution of Adyghe dominant dialects among the participants was as follows: Bzhedugh (*N* = 22), Kabardian (*N* = 12), Temirgoy (*N* = 11), and Abadzekh (*N* = 5).

It should be noted that Kabardian is treated among linguists as another Circassic language, − East Circassian (Daniel and Lander, 2011; Polinsky, 2020). At the same time, due to the great proximity and similarity to Adyghe, Kabardian speakers living in Maykop tend to identify themselves as Adyghe speakers of Kabardian variety, and point out that their Kabardian variety differs from the Kabardian language in the Republic of Kabardino-Balkaria. Hence, Kabardian participants were originally included in the present study, based on their self-identification, and on the language of their primary reading exposure – Temirgoy dialect in their former school education, with the latter being especially relevant for a reading study.

To ensure the homogeneity of the data sample, we checked for the differences in reading comprehension accuracy among the speakers of the four dialects. The Kruskal Wallis test (applied due to the non-normal distribution of residuals) showed that comprehension accuracy across the four dialects was different (chi-squared = 173.19, df = 2, *p* < 0.01). A post-hoc pairwise comparison of accuracies with a Dunn test confirmed that mean accuracies of Kabardian speakers differed significantly from speakers of Bzhedugh (adjusted *p* < 0.05) who represented the great majority of the population in Maykop, and of our dataset.

Based on the accuracy data differences, Kabardian speakers (*N* = 15) were excluded from further analysis. The final sample consisted, therefore, of 50 participants (44 women; Mean age = 32.7, SD = 14.1, range 18–60). The mean education level among participants was 15.1 years, SD = 2.1, range 11–20. We summarized the self-reported information about participants’ reading acquisition, reading skills, and reading exposure in both languages, from the shortened version of the Language Experience and Proficiency Questionnaire (LEAP-Q, Marian et al., 2007) in Table 1.

All participants had normal or corrected to normal vision. They all signed an informed consent form, and their participation was voluntary. The study was approved by the HSE Committee on Interuniversity Surveys and Ethical Assessment of Empirical Research.

### Materials and design

The materials of the study consisted of two corpora of sentences: The Russian Sentence Corpus (RSC, Laurinavichyute et al., 2019) and The Adyghe Sentence Corpus (ASC), which was compiled in an analogous way to the RSC. The first version of the ASC in 2021 included 60 sentences with word annotation, whereas 40 more sentences and target words for a more controlled study design were added later in 2022. Hence, the full version of the ASC included 100 sentences of different syntactic structures typical for Adyghe. Similarly to the RSC, all words in ASC were annotated for parts of speech, word frequency (retrieved from Adyghe Corpus),^3^ and word length. Apart from that, the ASC included morpheme annotation (the number of morphemes, and number of roots, number of grammatical and lexical affixes). The parts of speech annotation was performed according to the function of a word in a sentence instead of its actual belonging to a word class and contained bigger classes of words like VERB for all verb-based words including participles, or FUNCTION for all non-content words like prepositions, conjunctions, etc. The distribution of parts of speech in the ASC was as follows: Nouns 38.4%, Verbs 32%, Pronouns 6.7%, Function words 2.7%, Adjectives 8.6%, Adverbs 11.5%.

To enable an experimental design and control data analysis for frequency, word length, and parts-of-speech class, the Adyghe Sentence corpus included target words in eight conditions. The 2 × 2 × 2 design consisted of two parts-of-speech classes (nouns and verbs), two word length classes (short words of 1–7 characters and long words of 8–19 characters), and two word form frequency classes (low frequency < 10 items per million (ipm), high frequency > 20 ipm). Each condition was represented with eight target words in the middle of a sentence (i.e., not the first or the last word), resulting in 64 sentences with a target word. The description of both sentence corpora used in the study is provided in Table 2.

**Table 2:** Descriptive statistics of the two corpora: The Russian Sentence Corpus (taken from Laurinavichyute et al., 2019) and The Adyghe Sentence Corpus.

	The Russian Sentence Corpus (Laurinavichyute et al., 2019)	The Adyghe Sentence Corpus (This study)
Total number of sentences	144	100
Sentence length (in words)	Mean = 9 SD = 1.4 Range: 5–13	Mean = 6.7 SD = 1.8 Range: 2–11
Number of words	1,362 words 1,074 (without first and last words)	625 words 425 (without first and last words)
Word length (in characters)	Mean = 5.7 SD = 3 Range: 1–16	Mean = 7.5 SD = 3.8 Range: 1–28
Word form frequencies (item per million - ipm)	Class 1 (1–10 ipm) – 404 Class 2 (11–100 ipm) – 340 Class 3 (101–1,000) – 192 Class 4 (1,001 – 10,000) – 151 Class 5 (10,001 – max) – 131	Class 1 (1–10 ipm) – 240 Class 2 (11–100 ipm) – 153 Class 3 (101–1,000) – 128 Class 4 (1,001 – 10,000) – 64 Class 5 (10,001 – max) – 27 NA - 13

A comprehension question with multiple answer options followed 33% of Russian sentences and 40% of Adyghe sentences. An example of sentences with a question in both languages is provided in Table 3.

**Table 3:** Examples of stimuli in Russian (from RSC) and Adyghe (from ASC).

Stimuli	
Russian	
Sentence	*Взяв с собой фотоаппарат, вся семья поехала в парк на пикник.*
English translation	Taking a camera with them, the whole family went to a picnic in a park.
Glossing	Vsya-v s soboy fotoapparat, vsya semya poexa-l-a v park na piknik. Taking with themselves camera, the whole family went to park to a picnic.
Question	*Куда поехала семья на пикник?* Where did the family go?
Answer options	*В парк* *В лес* *В сад* To the park, to the forest, to the garden
Correct answer	*В парк* To the park
Adyghe	
Sentence	*ЧыжьэкIэ, псыхъом ушъхьэдэплъымэ, ордэ унашъхьэр къэлъагъощтыгъ.* far away river if you look across big rooftop could be seen
English translation	Far away, across the river, the roofs of an ancient castle could be seen.
Glossing	č-ẑe-č̣ʼje psəxo-m wə-ṣ̂xʰə-də-pʎə-m-ə ordə wənə-šxʰəxə-r čʼə-ʎa-ʁošʼ-təʁ
Question	*Сыда къэлъагъощтыгъэр?* What could be seen?
Answer options	*Ордэ унашъхь* *Ежь замокыр* The rooftop of an ancient castle, An ancient castle itself
Correct answer	*Ордэ унашъхь* The rooftop of an ancient castle

### Apparatus

Eye movements were recorded using an eye-tracking system EyeLink Portable Duo (SR Research, Canada), with sampling rate of 1,000 Hz. The stimuli were displayed in black Ubuntu Mono font, font size 30 pt., on a light-gray background of the ASUS ROG Zephyrus S GX701GV-EV006 laptop with 1920×1080 screen resolution and 144 Hz refresh rate. Participants were seated 52 cm from the screen, and 36.5 cm from the camera with their head positioned on a chin rest. Only the right eye was recorded.

### Procedure

**Figure 1:**
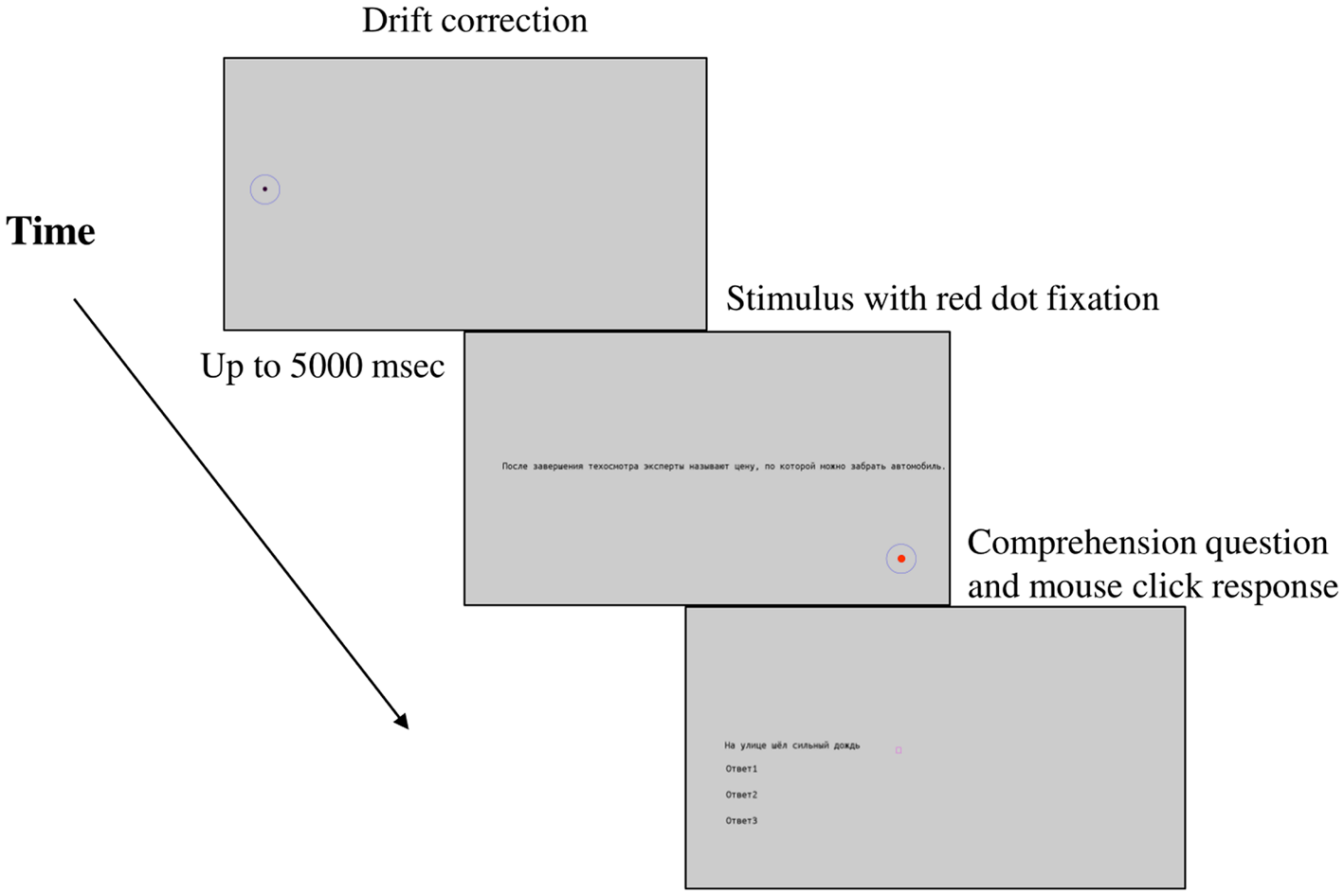
Experimental trial unfolding.

After signing a consent form, participants filled in a short questionnaire with their demographic data and their language background. Then, they proceeded with an eye-tracking part of the study. Participants from 2021 read the first version of the ASC with 60 sentences, whereas participants from 2022 read both corpora, in Russian and in Adyghe, in their final versions (i.e., 144 and 100 sentences respectively). In their case, the sequence of corpora presentations was counterbalanced. Participants were given both an oral and a written instruction about the experiment’s procedure. The eye-tracking-while-reading task started with a 9-point calibration (with an average error < = 0.5 and a maximum error < = 1.0), continued with the instruction for the experiment on the screen and was followed with the practice trials (5 in the RSC and 3 in ASC). Each trial started with a drift correction point on the position of the first letter in the first word of the sentence. If no fixation was detected within 500 msec, a recalibration was performed. Once a drift correction was successful, a sentence appeared in the middle of the screen. Participants were instructed to read sentences silently at their normal pace, and fixate on a red point in the right lower corner of the screen once they finished reading a sentence (see Figure 1 picturing how a trial was unfolding). After that, either a comprehension question, or a new trial appeared. Participants answered with a mouse click, choosing from the options presented. While reading one corpus, short breaks of 1–3 min were introduced, whereas a longer, up to 15 min break, was held between the two corpora. A re-calibration was performed after each break. Experimental procedure with.

### Data analysis

Statistical analysis was performed in R (R Core Team, 2020). Analysis of eye movements predominantly followed the protocol in Kliegl et al. (2006) and Laurinavichyute et al. (2019). Thus, the first and the last words in each sentence were removed. First fixation durations shorter than 60 ms were excluded from the analysis, as they were not likely to reflect lexical processing yet (see Sereno and Rayner, 2003). No upper cut-off limits were applied. The following 9 measurements of eye movements were chosen as dependent variables:

- first fixation duration (FFD);
- single fixation duration (SFD);
- Gaze duration (GD);
- total reading time (TT);
- probability of skipping the word (P0);
- probability of fixating the word only once (P1);
- Probability of fixating the word more than once (P2+);
- Probability of the word being an origin of a regressive saccade (RO);
- probability of the word being a goal of a regressive saccade (RG).

The listed measurements reflect both, early (FFD, SFD, GD, P0, P1) and late language processing (TT, P2+, RO, RG) - even though the same cognitive processes might overlap in different eye-movement measures (Holmqvist et al., 2011), early measures tend to be primarily associated with lexical activation, early information integration, and early morphological decomposition (Holmqvist et al., 2011, p. 385; Vasishth et al., 2013), while late measures reflect rather post-lexical processing including syntactic integration, and reanalysis (Boston et al., 2008; Holmqvist et al., 2011).

Continuous eye-movement outcome measures (FFD, SFD, GD, TT) were log-transformed and were fit with separate linear mixed-effects models. Binary variables (P0, P1, P2+, RO, RG) were fit with separate generalized linear mixed-effects models. Random effects for both model types included participants’ id, sentence number, and words. For modeling, lme4 package, version 1.1–31 (Bates et al., 2015) was used. Significant effects were adjusted for multiple comparisons with Bonferroni correction. Tables with the models’ output were created with sjPlot package, version 2.18.12 (Lüdecke, 2017), and are provided in the Supplementary materials. Figures were plotted with ggplot2 package, version 3.4.0 (Wickham, 2016).

The full list of independent variables was as follows:

- word frequency
- word length
- part-of-speech class (POS)
- word frequency of a previous word
- word frequency of a next word
- word length of a previous word
- word length of a next word
- word’s relative position in a sentence
- landing position (how far from the word beginning the first fixation landed)
- number of lexical affixes (for ASC only)
- self-reported reading skill score in Adyghe (for ASC only)

Following Laurinavichyute et al. (2019), all word frequencies were log-transformed, word length was centered, but not scaled. POS was a factor variable with 6 levels [VERB, NOUN, ADJ(ective), ADV(erb), PRONOUN, FUNCTION], with verbs being the basis for comparison. The number of lexical affixes, as well as the reading skill score were centered, but not scaled.

The data analysis of eye movements included two parts in line with the aims of the study. First, to establish benchmarks in eye movements while reading in Adyghe and report psycholinguistic features that affect reading in this language, we performed analysis of eye movements in ASC. This analysis included subparts of all-word analysis in the final sample of 50 participants, and target-word analysis in 38 participants from 2022 data collection (when target words were introduced in the final version of the ASC). Second, to disentangle the effect of language *per se* on reading in bilinguals with two morphologically different languages, we conducted a within-group analysis of eye movements on all words while reading two corpora: ASC and RSC (*N* = 38).

Thus, taking into account the different linear models depending on the eye-tracking measure in focus, and on the analysis type (all-word vs. target-word), there were several model structures. The full structure of the models for continuous eye-tracking measures in all-word analysis was as follows: *continuous eye-tracking measure ~ reading skill in Adyghe + word frequency + word length + next word’s length + next word’s frequency + previous word’s length + previous word’s frequency + word’s relative position + POS + number of lexical affixes + landing + (1 | participant) + (1 | sentence number) + (1 | word)*. The full structure of the models for binary eye-tracking measures in all-word analysis was as follows: *binary eye-tracking measure ~ reading skill in Adyghe + word frequency + word length + number of lexical affixes + (1 | participant) + (1 | sentence number) + (1 | word)*.

The full structure of the models in target-word analysis (for both continuous and binary eye-tracking measures) was shortened to the controlled independent variables only: *continuous/binary eye-tracking measure ~ word frequency + word length + POS + (1 | participant) + (1 | sentence number)*.

The full structure of the models for continuous eye-tracking measures in within-group analysis was as follows: continuous eye-tracking measure ~ lang*(reading skill in Adyghe + reading skill in Adyghe + word frequency + word length + next word’s length + next word’s frequency + previous word’s length + previous word’s frequency + word’s relative position + landing) + (1 | participant) + (1 | sentence number) + (1 | word). The full structure of the models for binary eye-tracking measures in within-group analysis was shortened to the very basic word features only: binary eye-tracking measure ~ lang*(word frequency + word length) + (1 | participant) + (1 | sentence number) + (1 | word). The code is freely available at Open Science Framework (OSF) platform, DOI 10.17605/OSF.IO/5UR8D.^4^

## Results

All model outputs with significant effects reported in this section (i.e., after Bonferroni correction) are provided in Supplementary Tables S1–S6.

### The benchmarks of eye movements in reading in Adyghe

The descriptive measures are summarized in Table 4 below.

**Table 4:** Descriptive statistics of eye-movements in reading ASC, Mean (SD).

Measure	Measurement	
FFD	msec	282.5 (48.12)
SFD		308 (50.4)
GD		662 (194)
TT		956 (301)
P0	%	1 (0.01)
P1		18 (0.08)
P2+		80 (0.08)
RO		23 (0.1)
RG		17 (0.08)
Fixation count	N	3.74 (1.01)
Landing position	%	31 (0.08)

#### Word frequency

**Figure 2:**
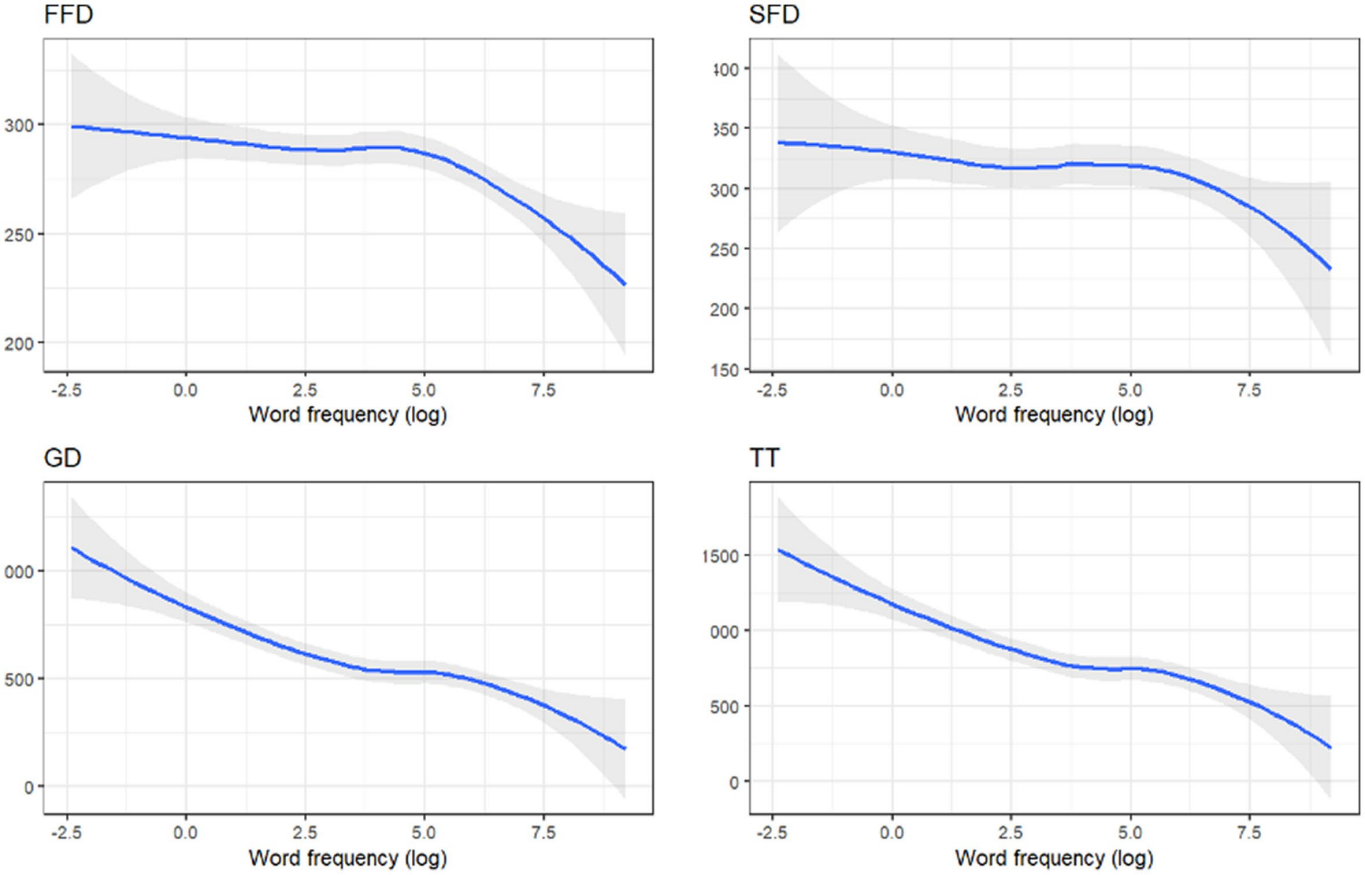
Estimated fixation durations depending on the word frequency.

A significant effect of a word form frequency was observed across all basic fixation duration measures: in FFD (Est. = −0.01, SE = 0.00, *t* = −3.90, *p* = 0.002), in SFD (Est. = −0.02, SE = 0.01, *t* = −3.47, *p* = 0.08), in GD (Est. = −0.03, SE = 0.00, *t* = −6.01, *p* < 0.001), and in TT (Est. = −0.03, SE = 0.01, t = −6.27, *p* < 0.001). The direction of the effect was as expected: the fixation duration decreased with a higher word form frequency as illustrated in Figure 2. More frequent words were significantly more likely to be fixated only once (P1: Log odds = 0.06, SE = 0.02, *t* = 3.20, *p* = 0.007), and were less likely to be fixated two or more times (P2+: Log odds = −0.07, SE = 0.02, *t* = −3.93, *p* < 0.001). Additionally, the probability of a word being a goal of regression decreased with higher word frequency (RG: Log odds = −0.05, SE = 0.02, *t* = −3.10, *p* = 0.01). In target word analysis, the more frequent words elicited longer fixation durations in TT only (Est. = −0.03, SE = 0.01, *t* = −3.17, *p* = 0.006).

#### Word length

**Figure 3:**
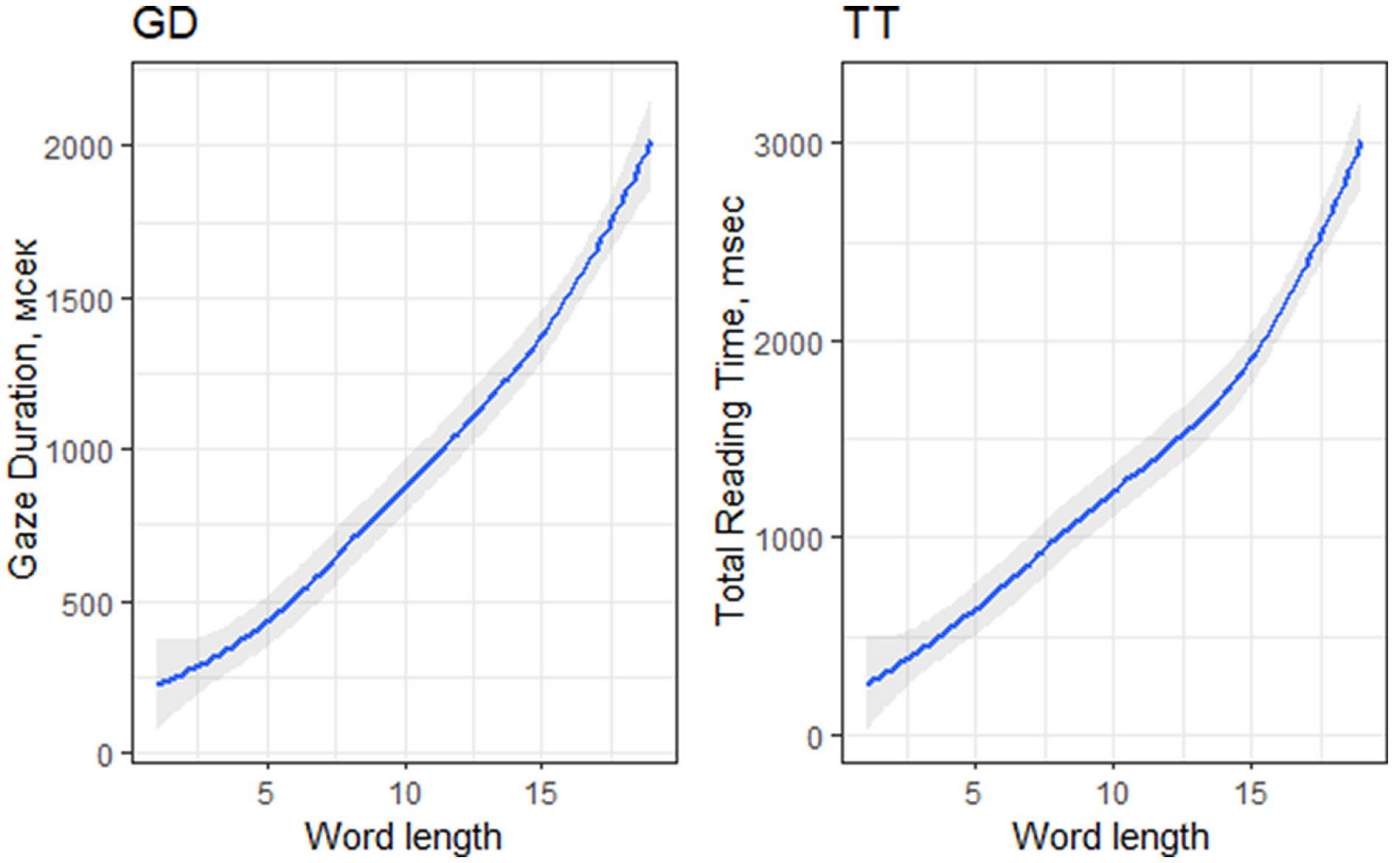
Word length effect on GD and TT in all-word analysis.

Longer words significantly increased GD (Est. = 0.10, SE = 0.00, *t* = 22.53, *p* < 0.001) and TT (Est. = 0.10, SE = 0.00, *t* = 20.66, *p* < 0.001) - see Figure 3. Longer words were shown to be less likely skipped (P0: Est. = −0.34, SE = 0.03, *t* = −10.38, *p* < 0.001) or fixated once only (P1: Est. = −0.50, SE = 0.02, *t* = −23.21, *p* < 0.001), whereas they were highly likely to be fixated more than twice (P2+: Est. = 0.54, SE = 0.02, *t* = 24.98, *p* < 0.001). Longer words were also significantly more likely to be a goal of regression (RG: Est. = −0.06, SE = 0.01, *t* = −4.75, *p* < 0.001).

In target word analysis, the longer words elicited longer fixation durations in GD (Est. = 0.10, SE = 0.01, *t* = 14.91, *p* < 0.001) and TT (Est. = 0.10, SE = 0.01, *t* = 16.22, *p* < 0.001). The effects of fixation probabilities remained stable: in P0 (Est. = −0.78, SE = 0.13, *t* = −5.98, *p* < 0.001), in P1 (Est. = −0.57, SE = 0.05, *t* = −12.33, *p* < 0.001), in P2+ (Est. = 0.60, SE = 0.05, *t* = 13.14, *p* < 0.001), and in RG (Est. = −0.08, SE = 0.03, *t* = −2.68, *p* = 0.029).

#### Morphological features: POS class and the number of lexical affixes

**Figure 4:**
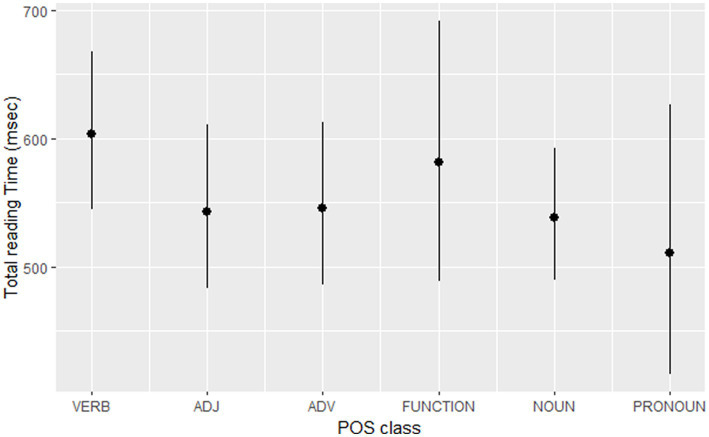
The predicted values of TT across parts of speech.

Nouns were read significantly faster than verbs (TT: Est. = −0.13, SE = 0.03, *t* = −4.20, *p* < 0.001). However, other POS did not differ significantly from verb reading. Moreover, the target-word analysis with verbs and nouns did not show significant effects of POS either. Figure 4 shows predicted values of total reading times across all parts of speech. The number of lexical affixes significantly increased TT (Est. = 0.20, SE = 0.06, *t* = 3.17, *p* = 0.025).

#### Word properties of previous and next words

Word length but not word frequency of a previous word significantly affected TT of reading a current word (Est. = −0.02, SE = 0.00, *t* = −3.92, *p* = 0.001). In turn, neither word length, nor word frequency of a next word affected eye movements while reading the current word.

#### Relative position and landing

**Figure 5:**
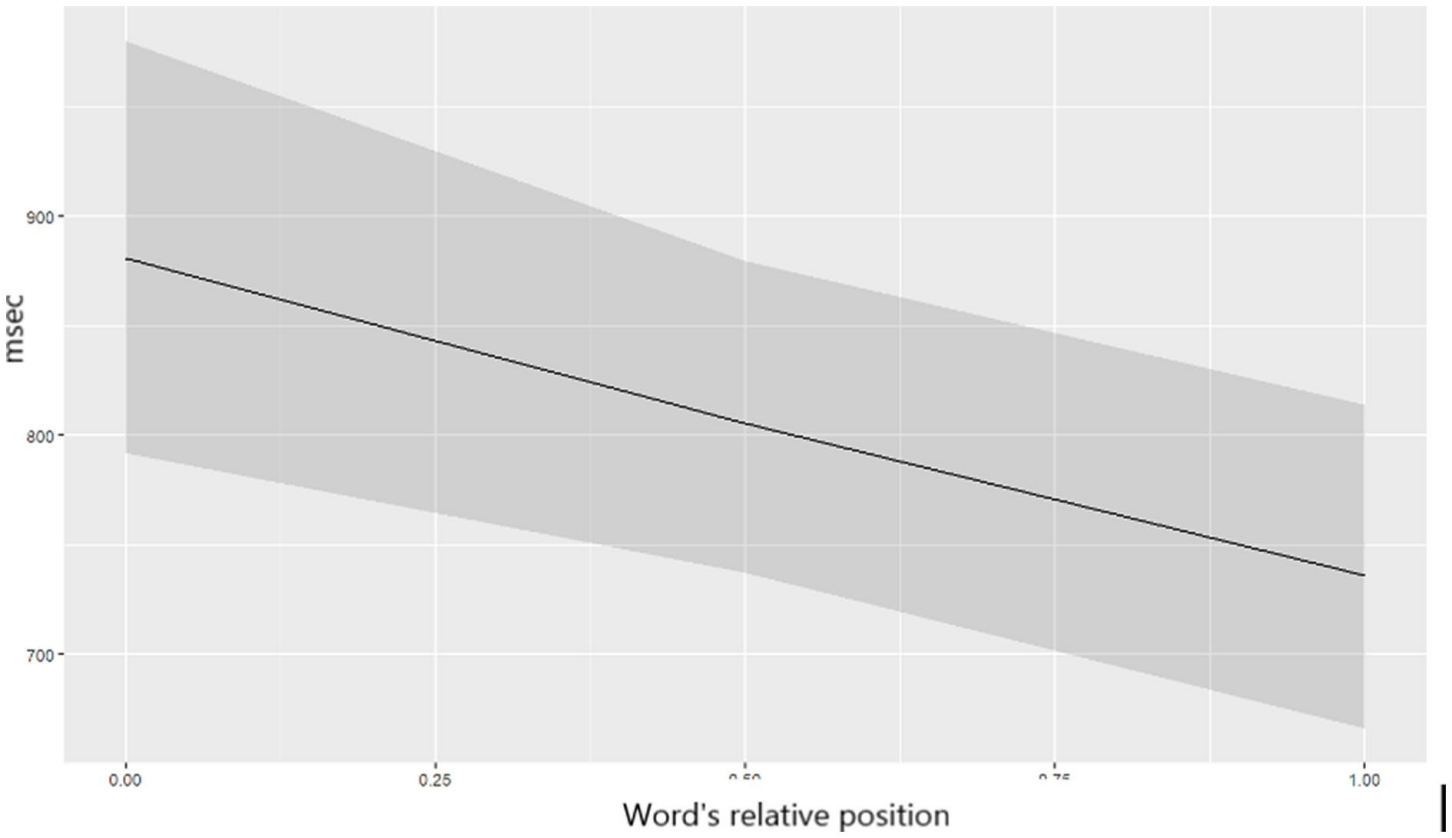
The predicted values of TT depending on the word’s relative position in a sentence.

**Figure 6:**
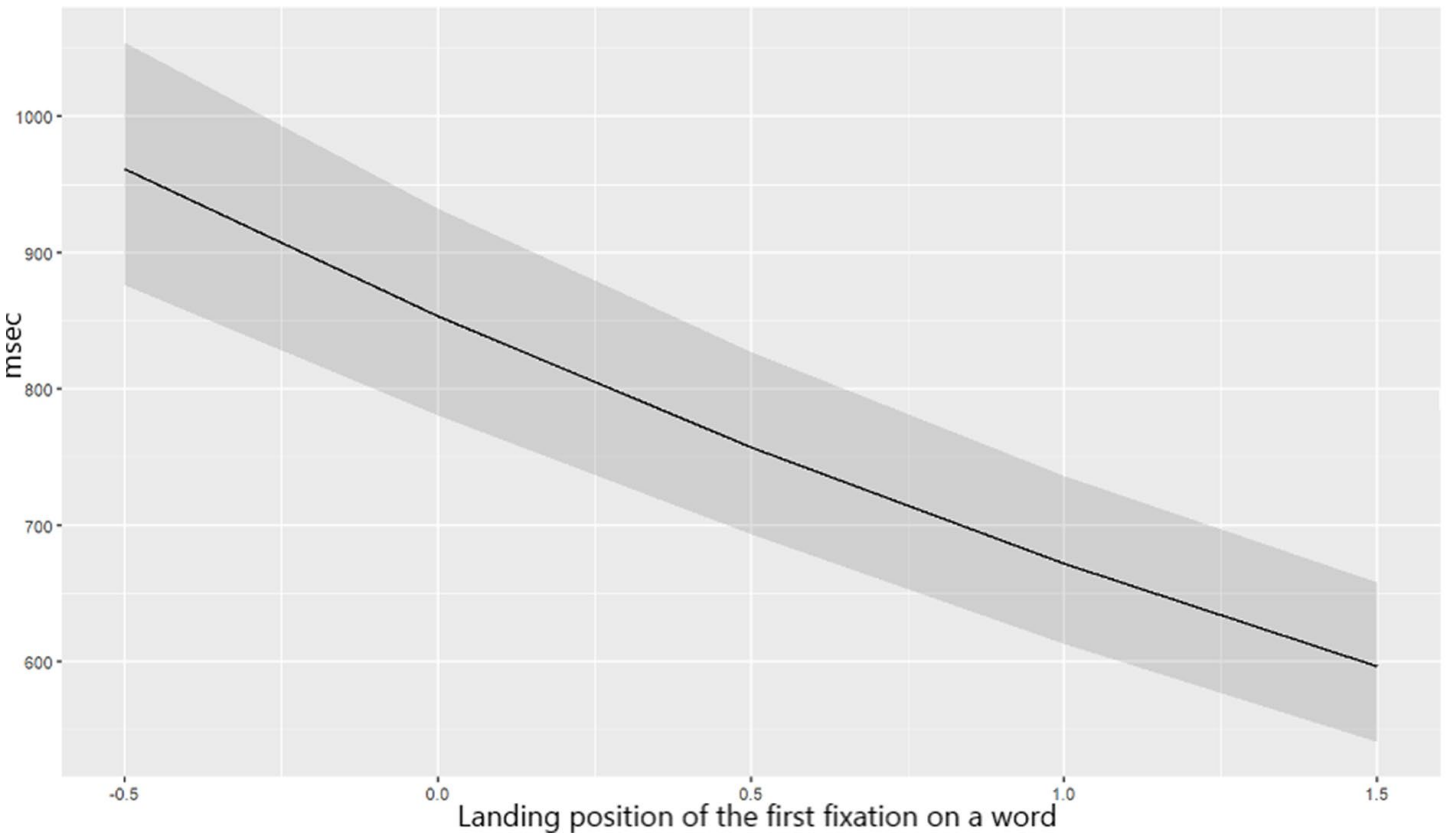
The predicted values of TT depending on the landing position of a first fixation on a word.

Words in the middle and closer-to-final positions were first fixated longer (seen in FFD increase: Est. = 0.08, SE = 0.03, *t* = 3.13, *p* = 0.028), but they were read significantly faster in total reading time than word in the initial positions (TT: Est. = −0.28, SE = 0.06, *t* = −4.98, *p* < 0.001 – see Figure 5). Landing position further from the word beginning elicited longer FFD (Est. = 0.22, SE = 0.02, *t* = 12.25, *p* < 0.001) and SFD (Est. = 0.09, SE = 0.03, *t* = 3.06, *p* = 0.036), whereas it shortened GD (Est. = −0.12, SE = 0.02, *t* = −5.42, *p* < 0.001) and TT (Est. = −0.23, SE = 0.02, *t* = −12.22, *p* < 0.001) (Figure 6).

#### Reading skill in Adyghe

The self-reported reading skill score in Adyghe significantly affected reading, which was seen in late fixation durations measures (GD and TT). With an increasing level of reading skills both measures decreased: GD with Est. = −0.16, SE = 0.04, *t* = −4.17, *p* < 0.001, and TT with Est. = −0.19, SE = 0.04, *t* = −4.52, *p* < 0.001.

### Within-group analysis of reading in two languages

To guarantee that a within-group analysis across two languages can be run, and reading in two languages is comparable in the group under study, we first analyzed reading comprehension in both languages. Comprehension accuracy in Russian was, on average, 0.9, SD = 0.07, range 0.69–1, and comprehension accuracy in Adyghe was, on average, 0.88, SD = 0.09, range 0.67–0.99. The Shapiro test showed that accuracy data distribution departed from normality (*p* < 0.001), which is why a non-parametric test was used. The Wilcoxon signed rank test resulted in non-significant differences between reading comprehension in both languages (*V* = 306, *p* < 0.5).

These results are essential for further data analysis and interpretation, as they validate a within-group comparison of reading in two languages, and eliminate the effect of longer reading times, due to poorer comprehension. Table 5 summarizes descriptive measures of eye movements while reading in two languages.

**Table 5:** Descriptive statistics of eye-movement measures in two languages (*N* = 38), Mean (SD).

Measure	Measurement	Adyghe	Russian
FFD	msec	290 (47.6)	211 (23.6)
SFD		317 (50.9)	226 (31.1)
GD		670 (167)	250 (37.7)
TT		936 (292)	300 (53.7)
P0	%	2 (0.02)	43 (0.1)
P1		20 (0.08)	40 (0.06)
P2+		77 (0.09)	16 (0.08)
RO		19 (0.09)	12 (0.07)
RG		17 (0.08)	12 (0.06)
Fixation count	N	3.52 (0.94)	0.82 (0.24)
Landing position	%	31 (0.07)	48 (0.04)

There were two significant effects observed consistently across all basic measures (FFD, SFD, GD, and TT): the effect of language (Adyghe), and word frequency. All reading times were higher in Adyghe compared to reading times in Russian (*p* < 0.001). Probability measures substantiated more effortful processing in Adyghe with lower probabilities of skipping and single fixations on words (both *p’s* < 0.001), and higher probabilities of 2+ fixations (*p* < 0.001) and regressions from the current word (*p* = 0.023).

A higher word frequency decreased all reading times (FFD, SFD, GD, and TT) with *p* < 0.001, increased skipping rate (*p* < 0.001), and decreased probabilities of more than one fixation (*p* < 0.001), re-fixations (*p* < 0.001), and regressions from the word (*p* = 0.012).

Word length and landing position were another two variables with consistent significant effects (*p* < 0.001) in SFD and GD. Additionally, an increased word length increased FFD (*p* = 0.003), TT (*p* < 0.001), and probability of more than one fixation (*p* < 0.001), whereas it decreased the probability of skipping and fixating a word once only (both *p’s* < 0.001). Landing position further from the word’s beginning increased not only SFD and GD, but also FFD (*p* < 0.001).

The main effects of parafoveal words (either frequency or length) were not significant. Word’s relative position further from the sentence beginning increased FFD (Est. = 0.03, SE = 0.01, *t* = 3.70, *p* = 0.005). The main effects of reading skills in Adyghe and Russian did not reach significance in any measures.

There were some interactions of language with other variables. We are reminded that Russian was taken as a baseline level for comparison, and it is, therefore, implied in the models’ intercept. Primarily, reading skills in both languages significantly affected reading in Adyghe, compared to reading in Russian, with *p* < 0.001 in all duration measures (FFD, SFD, GD, and TT). However, the direction of the effect was different. Higher reading skill in Adyghe accelerated reading in it compared to reading in Russian, whereas higher reading skills in Russian slowed down reading in Adyghe compared to reading in Russian.

No significant interaction of language and word frequency was found. Significant effects of word length in the interaction with language were found in late measures (GD and TT) and in fixation probabilities. Namely, longer words were read significantly longer in Adyghe (GD: Est. = 0.05, SE = 0.00, *t* = 10.45, *p* < 0.001; TT: Est. = 0.04, SE = 0.01, *t* = 7.19, *p* < 0.001) than words of the same length were read in Russian. Compared to Russian, longer words were less likely to be fixated only once (P1: Est. = −0.35, SE = 0.03, *t* = −13.54, *p* < 0.001), and were more likely to be fixated more than twice (P2+: Est. = 0.20, SE = 0.03, *t* = 6.83, *p* < 0.001).

The effects of the parafoveally located words’ properties (frequency and length) and current word’s relative position were not significant with the exception for the length of a previous word. Longer words on the left side decreased the total reading time of a current word (Est. = −0.02, SE = 0.00, *t* = −3.39, *p* = 0.015). Landing position further from the word’s beginning interacted with language in both early (FFD) and late measures (GD, TT). Namely, it took more time during the first fixation to process the word, and less time to read it in the next fixations compared to the same landing position in Russian.

## Discussion

The present study aimed to answer two research questions: (1) what are the benchmarks of eye movements while reading in a polysynthetic language (Adyghe), and (2) how does its reading differ from reading in a synthetic language (Russian) that is based on the same script? To answer these questions, we collected eye-movement data while reading two corpora: the Russian Sentence Corpus (RSC, Laurinavichyute et al., 2019) and the Adyghe Sentence Corpus (ASC). The analysis of eye movements included two parts in line with the research questions. First, an analysis (of all words and target words exclusively) in a larger data sample (*N* = 50) while reading ASC was performed. Second, we conducted a within-group analysis (*N* = 38) of eye movements comparing reading in two languages.

### Benchmarks of eye movements while reading in Adyghe

Overall, the most robust universal effects of word frequency and word length on eye movements found in previous research across different languages (Inhoff and Rayner, 1986; Rayner, 1998; Staub and Rayner, 2007) were confirmed in our study in a polysynthetic language. Simultaneously, the finding of word frequency not being a significant effect across a range of measures contradicts the previously studied effects across languages and might imply some inconsistencies in the Adyghe Corpus, which is a constantly developing source of word frequencies in Adyghe. Presumably, the word frequencies extracted at the moment of the study did not fully reflect actual language use and might need to be updated.

On the other hand, this peculiarity brings us to the underlying question of the definition of a word and its units in polysynthetic languages. Lexical affixes might be confused with roots, and a word form reflects not just the form variations of a lemma but new “words” in its common notion. The blurred word boundaries (Haspelmath, 2018) make it possible that we need a shift toward other frequency measures. It will likely be more efficient to include morpheme frequency and/or initial bigram frequency similar to the analysis conducted in Yan et al. (2014) in Uighur.

We also confirmed another universal effect in eye-tracking-while-reading research - the effect of word length. It was consistently observed in late duration measures, as well as in probability measures. Importantly, no effect of word length in early measures (FFD and SFD) resembles reading in Russian among monolingual adults in Laurinavichyute et al. (2019) and HSs in Parshina et al. (2021). No effect in RO and lower probability of regressions to the longer word (RG) are compatible with those in German (Kliegl et al., 2006), but not in Russian (Laurinavichyute et al., 2019; Parshina et al., 2021).

The effects of a previous/upcoming word in Adyghe partially resemble those in German monolinguals (Kliegl et al., 2006) and in high proficient Russian HSs (Parshina et al., 2021) but not in Russian monolinguals (Laurinavichyute et al., 2019). Specifically, longer previous words accelerated the total reading time of a current word in Adyghe, whereas longer upcoming words did not show any effect. This outcome seems logical, taking into account the higher average word length in Adyghe (*cf.* 7.5 letters in ASC vs. 5.7 letters in RSC) which does not enable their readers to extract lexical information from the right side in the parafoveal processing.

The influence of POS class on eye movements in Adyghe was in line with previous research in Russian (Laurinavichyute et al., 2019): verbs were read significantly slower than nouns (in TT), whereas other POS did not differ significantly from verb reading. This finding corresponds to the notion, across different fields of linguistics, that verbs are more complex units and are more difficult to acquire and process than nouns (Bassano, 2000; Mätzig et al., 2009; Crepaldi et al., 2011).

Finally, we observed a morphological effect of lexical affixes on eye movements in a polysynthetic language. Essentially, this finding confirmed that a higher number of lexical affixes increases cognitive load and is a relevant lexical feature to be controlled for. However, we have to acknowledge its limited distribution: only total reading times in all-word analysis, but not in target-word analysis, were affected. Presumably, either the limited distribution of the effect in a sentence or less controlled materials might account for these results.

A limited distribution of the morphological effect (monomorphemic vs. inflected words) was earlier observed in agglutinative languages (Finnish and Turkish). In Finnish, isolated words were affected by morphological complexity, whereas words in a sentence context were not (Hyönä et al., 2002). In Turkish, this effect in sentence reading was observed in probability measures but not in early measures like SFD (Özkan et al., 2021). Having said that, a preserved effect of morphological complexity in a sentence context was reported in Yan et al. (2014) on the materials of a highly agglutinative language (Uighur) in both early (FFD) and late (GD) measures.

### Reading in polysynthetic Adyghe vs. reading in synthetic Russian

Whereas reading in both languages seem to be affected similarly by word frequency with more frequent words being read faster, it seems to be affected differently by word length. Namely, two significant interactions of word length with language while reading in Adyghe demonstrated that longer words in Adyghe are read slower than words of the same length in Russian. This might account either for morphological differences between languages (longer Adyghe words might have a more complex morphemic structure which loads processing, whereas long Russian words are not necessarily polymorphemic) or for differences in participants’ reading skills in the two languages.

The opposite effects of reading skills in Adyghe and Russian during reading in Adyghe reflect a common debate regarding language interference in bilinguals (Kaushanskaya and Marian, 2007; Libben and Titone, 2009). A higher reading skill in one language accelerated processing in that language but inevitably impeded processing in another one. Hence, participants with a higher self-assessed reading skill in Adyghe read Adyghe sentences faster than Russian ones, whereas participants with a higher self-assessed reading skill in Russian read Adyghe sentences slower than Russian ones.

Non-significant main effects of the neighboring words characteristics (frequency and length) together with their non-significant interactions with language leads us to conclude that speakers of a polysynthetic language do not rely on information about neighboring words. On the contrary, different Russian-speaking groups (monolinguals in Laurinavichyute et al., 2019; HSs and L2 learners in Parshina et al., 2021) do extract some information from upcoming words, even though it is predominantly observed on late measures. Apparently, bilingual speakers of a polysynthetic language transfer this processing pattern to their other language (Russian, in this case), which distinguishes their reading in Russian from other Russian-speaking populations.

Noteworthy are the differences in the preferred landing position across the two languages. Statistical analysis showed that a further landing position on an Adyghe word will result in more efficient word processing (with a longer FFD but shorter GD and TT) compared to reading in Russian if landing on the same position. Descriptively, Adyghe bilinguals tend to land closer to the word beginning (on the first 31% of the word letters) when reading in Adyghe and closer to the word’s center (on the first 48% of the word letters) when reading in Russian.

### Limitations and further research

We must admit some limitations of the study. A corpus study has the pitfall of using less controlled materials, which can lead to multicollinearity among predictors. We partially addressed this issue in the target-word analysis, where three variables were controlled (frequency, length, and POS with verbs and nouns as levels), and in the all-word analysis, where the variance of the inflation factor (VIF) of the predictors was always less than 2. Most morphemic features in our data (except for the number of lexical affixes) were highly correlated with word length, which restricted us to one morphemic variable in the analysis and limited our investigation of morphological effects on reading in Adyghe.

Consequently, the primary suggestion for further research is either an orthogonally-designed experimental study on reading in Adyghe or a further exploitation of the ASC from a different perspective. For instance, the number of morphemes, together with the number of lexical and grammatical affixes, could be considered for another controlled-condition study. The great variety of dialects in Adyghe is an area for further corpus research. Not only was dialectal variation not the focus of our study, but we also had to exclude speakers of Kabardian from the analysis to ensure comparability with other dialects. Their data are, in turn, freely available together with other materials of the study at OSF, DOI 10.17605/OSF.IO/5UR8D (see footnote 4) and can be used in further research.

Apart from that, we see a potential to investigate in more detail the transfer of reading patterns that bilinguals make from one language to another. In our study, we observed this kind of transfer regarding the neighboring words: Adyghe-Russian bilinguals do not rely on their characteristics while reading in any language, whereas other Russian-speaking populations do when they read in Russian. The list of independent variables used to study eye movements from this perspective could be extended, and a different type of analysis (e.g., a scanpath analysis) could shed more light on reading patterns in the two languages and their interaction.

## Data availability statement

The datasets presented in this study can be found in online repositories. The names of the repository/repositories and accession number(s) can be found below: https://osf.io/5ur8d/?view_only=432e327cd0e64b5ca062be7e7e56b9b3.

## Ethics statement

The studies involving humans were approved by the HSE Committee on Interuniversity Surveys and Ethical Assessment of Empirical Research. The participants provided their written informed consent to participate in this study.

## Author contributions

OD and OP contributed to the conception of the study, curatorship of the data collection, and manuscript editing. NZ, OP, BO, IB, ShU, and SM were responsible for the stimuli creation. NZ, OP, BO, EK, AZ, and SM contributed to the participants recruitment and data collection. NZ contributed to the coding, data analysis, manuscript writing and editing. All authors contributed to the article and approved the submitted version.

## Funding

This article is an output of a research project implemented as part of the Basic Research Program at the National Research University Higher School of Economics (HSE University).

## Conflict of interest

The authors declare that the research was conducted in the absence of any commercial or financial relationships that could be construed as a potential conflict of interest.

## Publisher’s note

All claims expressed in this article are solely those of the authors and do not necessarily represent those of their affiliated organizations, or those of the publisher, the editors and the reviewers. Any product that may be evaluated in this article, or claim that may be made by its manufacturer, is not guaranteed or endorsed by the publisher.

## Footnotes

^1^In this paper, we stick to the term Adyghe that is widely used in typological literature, including The World Atlas of Language Structures (WALS, Dryer and Haspelmath, 2013).

^2^ https://eng.rosstat.gov.ru/

^3^ http://adyghe.web-corpora.net/

^4^ https://osf.io/5ur8d/?view_only=432e327cd0e64b5ca062be7e7e56b9b3

## References

[ref1002] Arkhangelskiy T. Bagirokova I. Lander Y. Lander A. Adyghe Corpus. Available at: http://adyghe.web-corpora.net/index_en.html (Accessed April 26, 2023).

[ref1] Bassano D. (2000). Early development of nouns and verbs in French: exploring the interface between lexicon and grammar. J. Child Lang. 27, 521–559. doi: 10.1017/S0305000900004396, PMID: 11089338

[ref2] Bates D. Mächler M. Bolker B. Walker S. (2015). Fitting linear mixed-effects models using lme4. J. Stat. Softw. 67, 1–48. doi: 10.18637/jss.v067.i01

[ref3] Boston M. Hale J. Kliegl R. Patil U. Vasishth S. (2008). Parsing costs as predictors of reading difficulty: an evaluation using the Potsdam sentence Corpus. J. Eye Mov. Res. 2, 1–12. doi: 10.16910/jemr.2.1.1

[ref4] Clifton C. Jr. Staub A. Rayner K. (2007). “Eye movements in reading words and sentences” in Eye movement research: Insights into mind and brain. eds. Gompel R. V. Fisher M. Murray W. Hill R. L. (New York: Elsevier), 341–371.

[ref5] Cop U. Dirix N. Drieghe D. Duyck W. (2017). Presenting GECO: an eyetracking corpus of monolingual and bilingual sentence reading. Behav. Res. 49, 602–615. doi: 10.3758/s13428-016-0734-0, PMID: 27193157

[ref6] Cop U. Drieghe D. Duyck W. (2015). Eye movement patterns in natural reading: a comparison of monolingual and bilingual reading of a novel. PLoS One 10:e0134008. doi: 10.1371/journal.pone.0134008 26287379 PMC4545791

[ref7] Crepaldi D. Berlingeri M. Paulesu E. Luzzatti C. (2011). A place for nouns and a place for verbs? A critical review of neurocognitive data on grammatical-class effects. Brain Lang. 116, 33–49. doi: 10.1016/j.bandl.2010.09.005 21036389

[ref8] Daniel M. Lander Y. (2011). “The Caucasian languages” in The languages and linguistics of Europe. A comprehensive guide. eds. Kortmann B. Auwera J. V., vol. 1 (Berlin, Boston: De Gruyter Mouton), 125–157.

[ref9] Data and data analysis at Open Science Framework. Available at: https://osf.io/5ur8d/?view_only=432e327cd0e64b5ca062be7e7e56b9b3

[ref10] Dryer M. S. Haspelmath M. (2013). WALS Online (v2020.3) [Data set]. Zenodo. Available at: https://wals.info (Accessed April 26, 2023).

[ref11] Haspelmath M. (2018). The last word on polysynthesis: a review article. Linguist. Typol. 22, 307–326. doi: 10.1515/lingty-2018-0011

[ref12] Holmqvist K. Nyström N. Andersson R., (2011). in Dewhurst R. Jarodzka H. Van de Weijer J. (Eds.) Eye tracking: A comprehensive guide to methods and measures, Oxford, UK: Oxford University Press.

[ref13] Hyönä J. Vainio S. Laine M.. (2002). A morphological effect obtains for isolated words but not for words in sentence context. Eur. J. Cogn. Psychol. 14, 417–433. doi: 10.1080/09541440143000131

[ref14] Inhoff A. W. Radach R. (1998). “Definition and computation of oculomotor measures in the study of cognitive processes” in Eye guidance in reading and scene perception. ed. Underwood G. (Elsevier Science Ltd.), 29–53.

[ref15] Inhoff A. W. Rayner K. (1986). Parafoveal word processing during eye fixations in reading: effects of word frequency. Percept. Psychophys. 40, 431–439. doi: 10.3758/BF03208203 3808910

[ref16] Kaushanskaya M. Marian V. (2007). Bilingual language processing and interference in bilinguals: evidence from eye tracking and picture naming. Lang. Learn. 57, 119–163. doi: 10.1111/j.1467-9922.2007.00401.x

[ref17] Kliegl R. Grabner E. Rolfs M. Engbert R. (2004). Length, frequency, and predictability effects of words on eye movements in reading. Eur. J. Cogn. Psychol. 16, 262–284. doi: 10.1080/09541440340000213

[ref18] Kliegl R. Nuthmann A. Engbert R. (2006). Tracking the mind during reading: the influence of past, present, and future words on fixation durations. J. Exp. Psychol. Gen. 135, 12–35. doi: 10.1037/0096-3445.135.1.12 16478314

[ref19] Laurinavichyute A. K. Sekerina I. A. Alexeeva S. Bagdasaryan K. Kliegl R. (2019). Russian sentence Corpus: benchmark measures of eye movements in reading in Russian. Behav. Res. Methods 51, 1161–1178. doi: 10.3758/s13428-018-1051-6 29907908

[ref20] Libben M. R. Titone D. A. (2009). Bilingual lexical access in context: evidence from eye movements during reading. J. Exp. Psychol. Learn. Mem. Cogn. 35, 381–390. doi: 10.1037/a0014875 19271853

[ref21] Liversedge S. P. Drieghe D. Li X. Yan G. Bai X. Hyönä J. (2016). Universality in eye movements and reading: a trilingual investigation. Cognition 147, 1–20. doi: 10.1016/j.cognition.2015.10.013 26605961

[ref22] Lopukhina A. Zdorova N. Staroverova V. Ladinskaya N. Kaprielova A. Goldina S.. (2022). Benchmark measures of eye movements during reading in Russian children. PsyArXiv [Preprint]. doi: 10.31234/osf.io/2x5pk

[ref23] Lüdecke D. (2017). sjstats: statistical functions for regression models. R package version 0.12.0. Available at: https://CRAN.R-project.org/package=sjstats

[ref24] Marian V. Blumenfeld H. K. Kaushanskaya M. (2007). The language experience and proficiency questionnaire (LEAP-Q): assessing language pro-files in bilinguals and multilinguals. J. Speech Lang. Hear. Res. 50, 940–967. doi: 10.1044/1092-4388(2007/067) 17675598

[ref25] Mätzig S. Druks J. Masterson J. Vigliocco G. (2009). Noun and verb differences in picture naming: past studies and new evidence. Cortex 45, 738–758. doi: 10.1016/j.cortex.2008.10.003 19027106

[ref26] Özkan A. Beken Fikri F. Kırkıcı B. Kliegl R. Acartürk C. (2021). Eye movement control in Turkish sentence reading. Q. J. Exp. Psychol. 74, 377–397. doi: 10.1177/1747021820963310 32976053

[ref27] Parshina O. Laurinavichyute A. Sekerina I. (2021). Eye-movement benchmarks in heritage language reading. Biling. Lang. Congn. 24, 69–82. doi: 10.1017/S136672892000019X

[ref28] Polinsky M. (ed.) (2020). “Introduction” in The Oxford handbook of languages of the Caucasus. (Oxford: Oxford University Press), 1–25.

[ref29] R Core Team (2020). R: A language and environment for statistical computing. R Foundation for Statistical Computing, Vienna, Austria. Available at: https://www.r-project.org/

[ref30] Rakhlin N. V. Kornilov S. A. Grigorenko E. L. (2017). “Learning to read Russian” in Learning to read across languages and writing systems. eds. Verhoeven L. Perfetti C. (Cambridge University Press), 393–415.

[ref31] Rayner K. (1998). Eye movements in reading and information processing: 20 years of research. Psychol. Bull. 124, 372–422. doi: 10.1037/0033-2909.124.3.372 9849112

[ref32] Rayner K. Well A. D. (1996). Effects of contextual constraint on eye movements in Reading: a further examination. Psychon. Bull. Rev. 3, 504–509. doi: 10.3758/BF03214555 24213985

[ref33] Rothman J. Bayram F. DeLuca V. di Pisa G. Duñabeitia J. Gharibi K.. (2022). Monolingual comparative normativity in bilingualism research is out of “control”: arguments and alternatives. Appl. Psycholinguist. 44, 316–329. doi: 10.1017/S0142716422000315

[ref1020] Russian Population Census (2020). Available at: https://eng.rosstat.gov.ru/ (Accessed April 26, 2023).

[ref34] Schilling H. H. Rayner K. Chumbley J. I. (1998). Comparing naming, lexical decision, and eye fixation times: word frequency effects and individual differences. Mem. Cogn. 26, 1270–1281. doi: 10.3758/bf03201199 9847550

[ref35] Schmauder A. R. Morris R. K. Poynor D. V. (2000). Lexical processing and text integration of function and content words: evidence from priming and eye fixations. Mem. Cogn. 28, 1098–1108. doi: 10.3758/BF03211811 11126934

[ref36] Sereno S. C. Rayner K. (2003). Measuring word recognition in reading: eye movements and event-related potentials. Trends Cogn. Sci. 7, 489–493. doi: 10.1016/j.tics.2003.09.010 14585445

[ref37] Siegelman N. Schroeder S. Acartürk C. Ahn H. D. Alexeeva S. Amenta S.. (2022). Expanding horizons of cross-linguistic research on reading: the multilingual eye-movement Corpus (MECO). Behav Res 54, 2843–2863. doi: 10.3758/s13428-021-01772-6, PMID: 35112286 PMC8809631

[ref38] Staub A. Rayner K. (2007). “Eye movements and on–line comprehension processes” in The Oxford handbook of psycholinguistics. ed. Gaskell M. G. (Oxford: Oxford University Press), 325–342.

[ref39] Sui L. Dirix N. Woumans E. Duyck W. (2022). GECO-CN: Ghent eye-tracking Corpus of sentence reading for Chinese-English bilinguals. Behav. Res. doi: 10.3758/s13428-022-01931-3 35896891

[ref40] Vasishth S. von der Malsburg T. Engelmann F. (2013). What eye movements can tell us about sentence comprehension. WIREs Cogn. Sci. 4, 125–134. doi: 10.1002/wcs.1209 26304190

[ref41] Wickham H. (2016). ggplot2: Elegant graphics for data analysis. Springer-Verlag New York. Available at: https://ggplot2.tidyverse.org.

[ref42] Yan M. Zhou W. Shu H. Yusupu R. Miao D. Krügel A.. (2014). Eye movements guided by morphological structure: evidence from the Uighur language. Cognition 132, 181–215. doi: 10.1016/j.cognition.2014.03.008 24813572

[ref43] Zhukova M. Grigorenko E. (2019). “Developmental dyslexia in Russia” in Developmental dyslexia across languages and writing systems. eds. Verhoeven L. Perfetti C. Pugh K. (Cambridge University Press), 133–151.

[ref1003] Zdorova N. (2023). Data and data analysis at Open Science Framework. [Data set]. Available at: https://osf.io/5ur8d/?view_only=432e327cd0e64b5ca062be7e7e56b9b3, PMID: 11089338

