# Supplementary material for: Eye movement corpora in Adyghe and Russian: an eye-tracking study of sentence reading in bilinguals
Source: Front Psychol. 2023 Sep 13;14:1212701. doi: 10.3389/fpsyg.2023.1212701 (PMC10534991; doi:10.3389/fpsyg.2023.1212701)

**Supplementary Materials**

Table 1. Model estimates of fixation durations in all-word analysis of ASC.


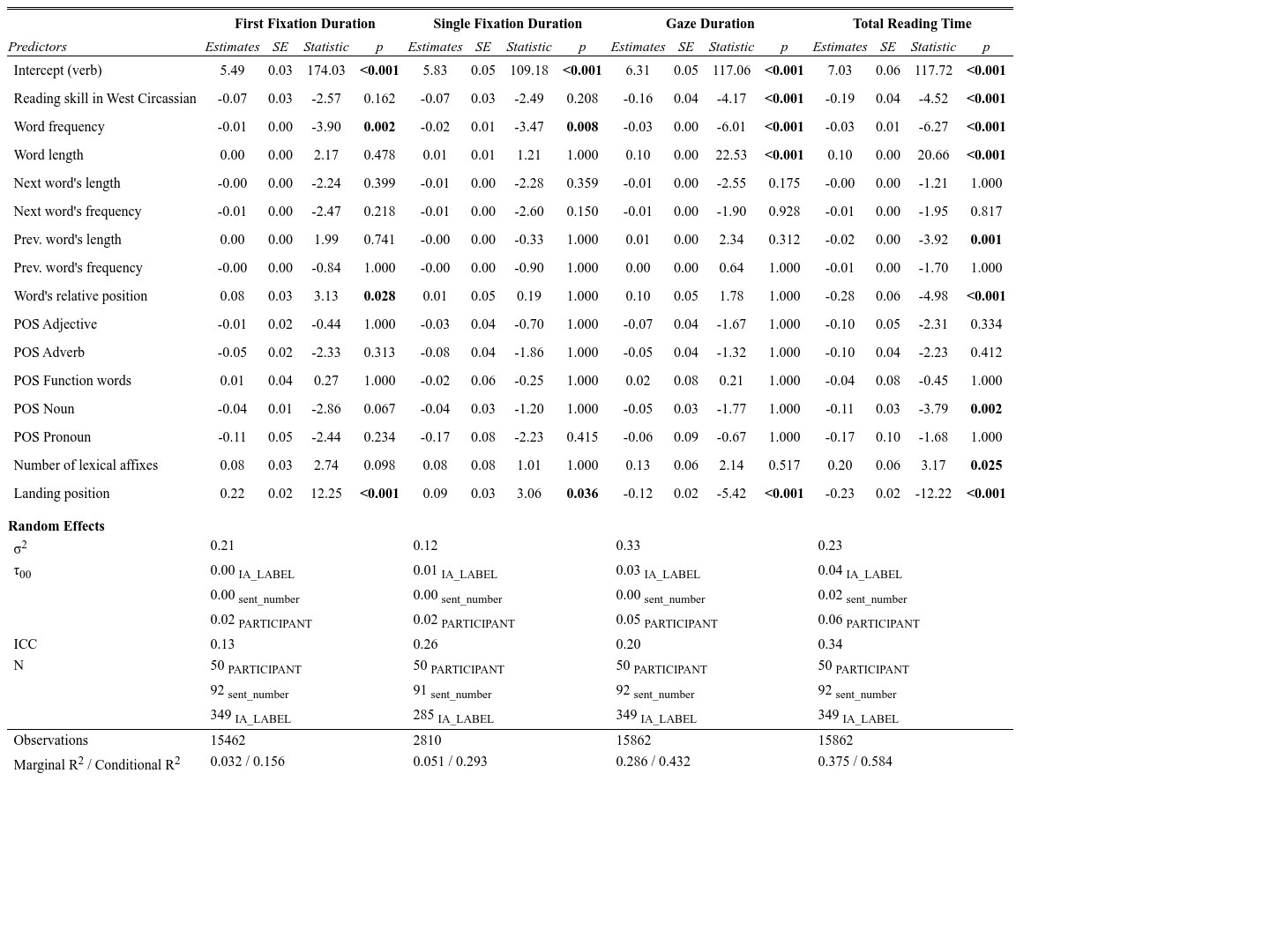


Table 2. Model estimates of probability measures in all-word analysis of ASC.


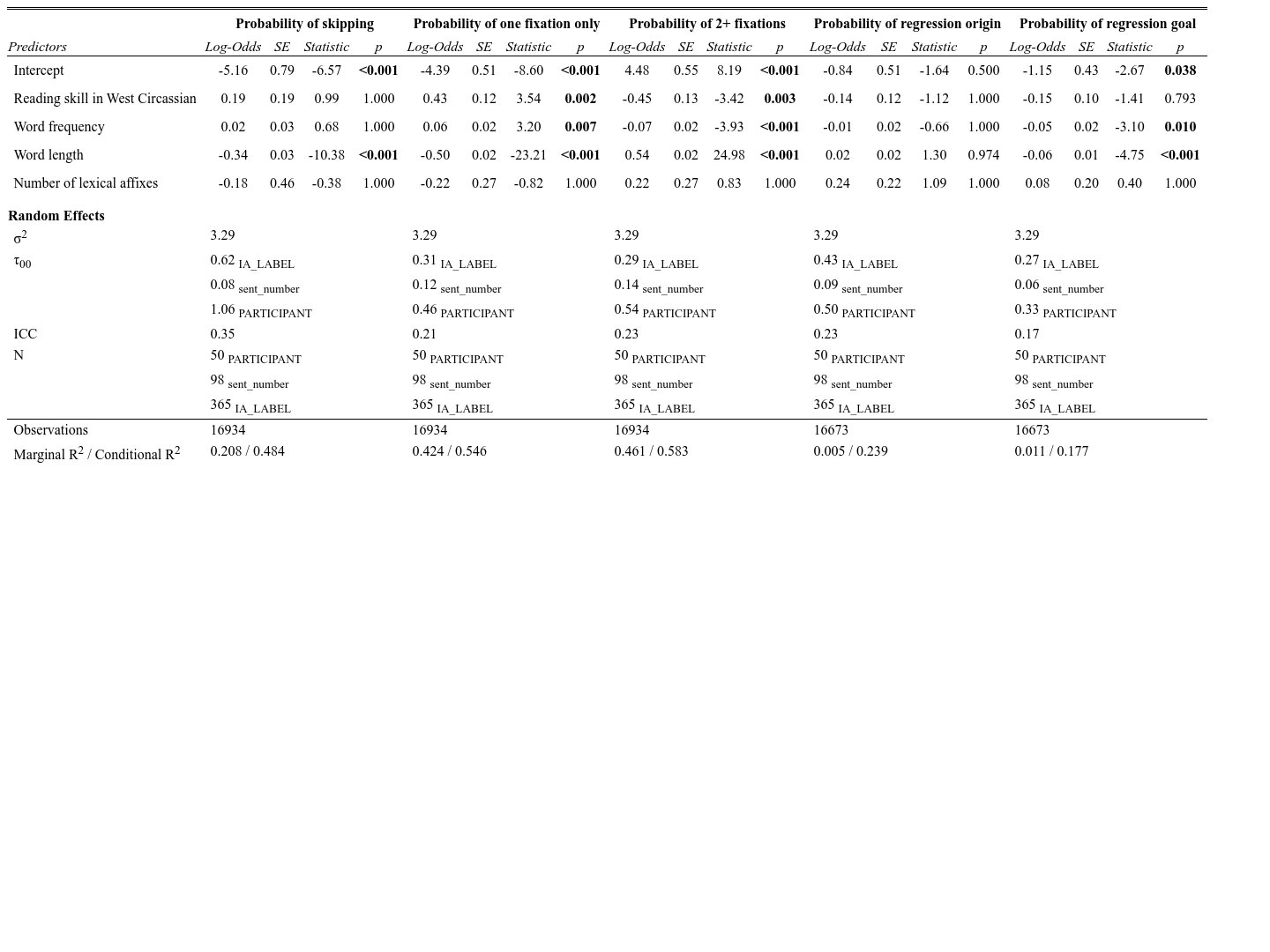


Table 3. Model estimates of fixation durations in target-word analysis of ASC.


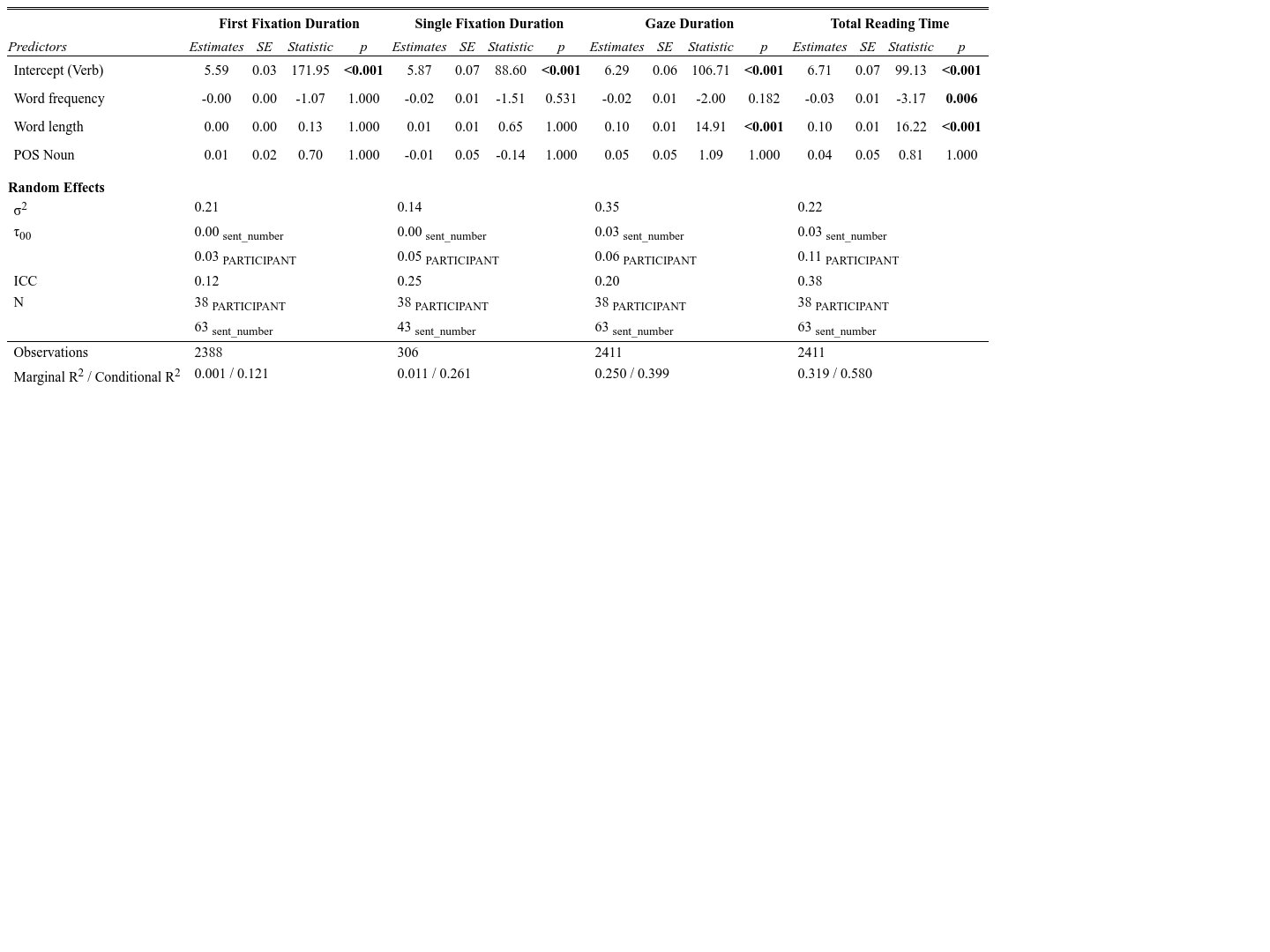


Table 4. Model estimates of probability measures in target-word analysis of ASC.


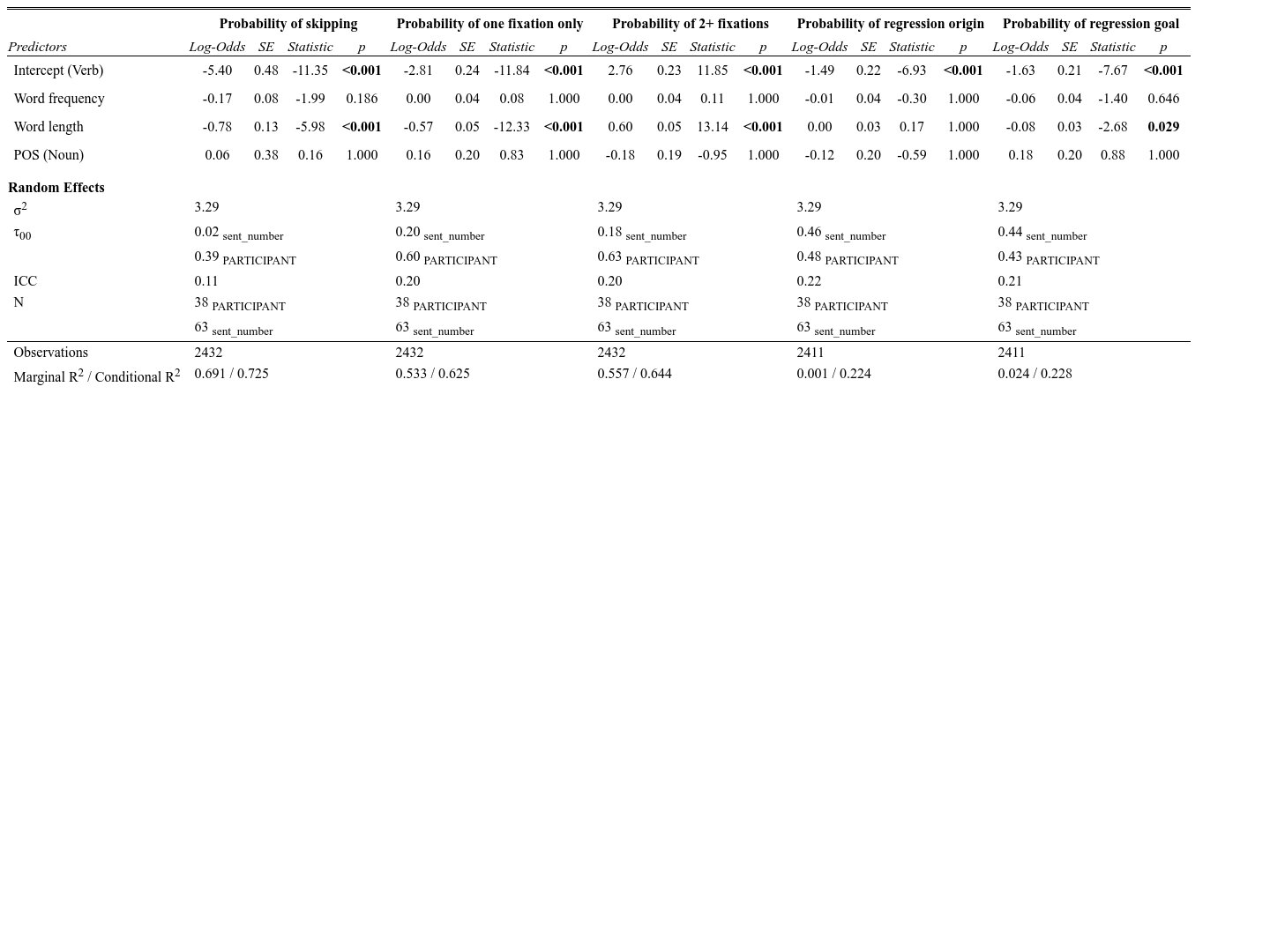


Table 5. Model estimates of fixation durations in within-group analysis.


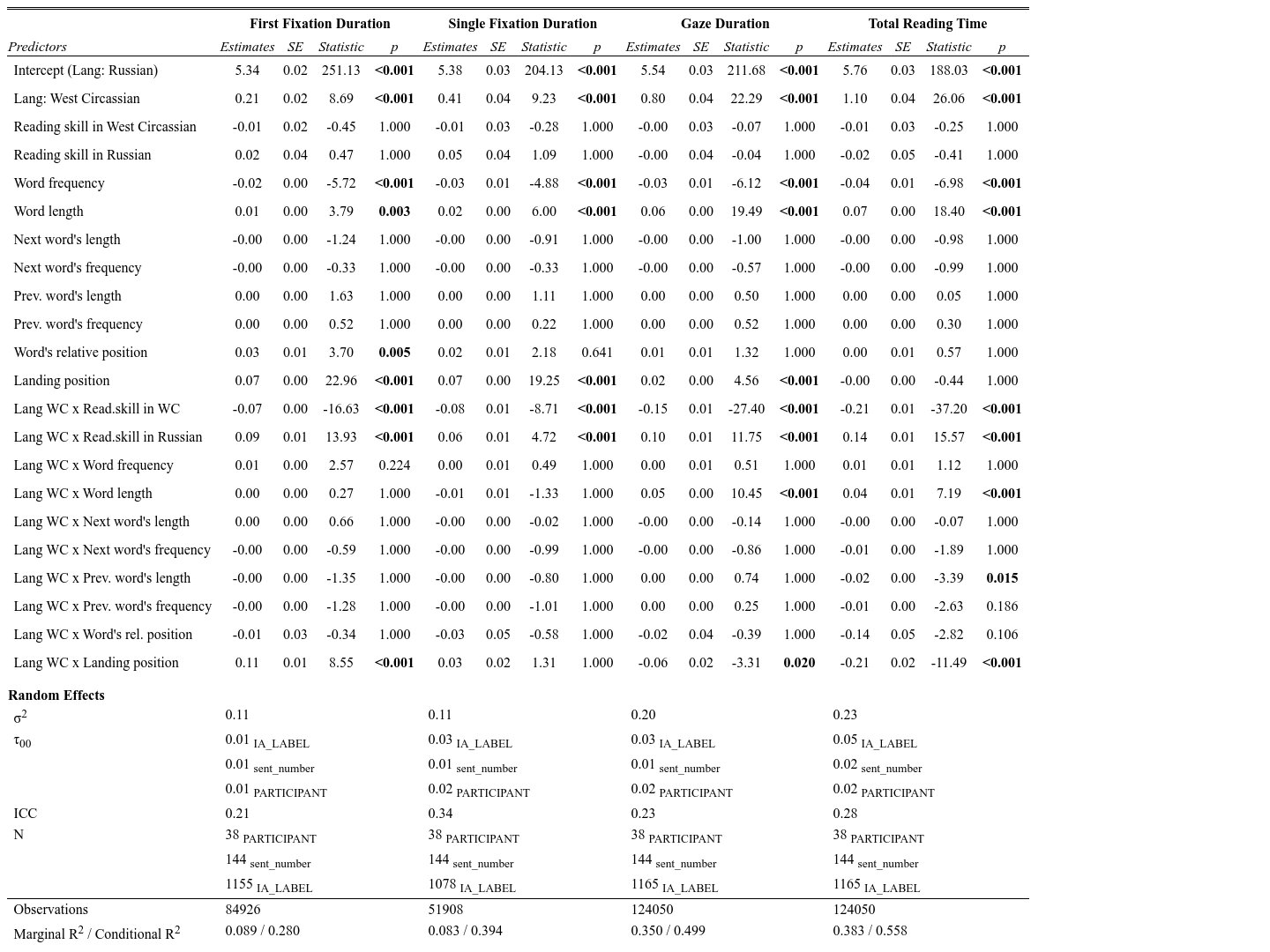


Table 6. Model estimates of probability measures in within-group analysis.
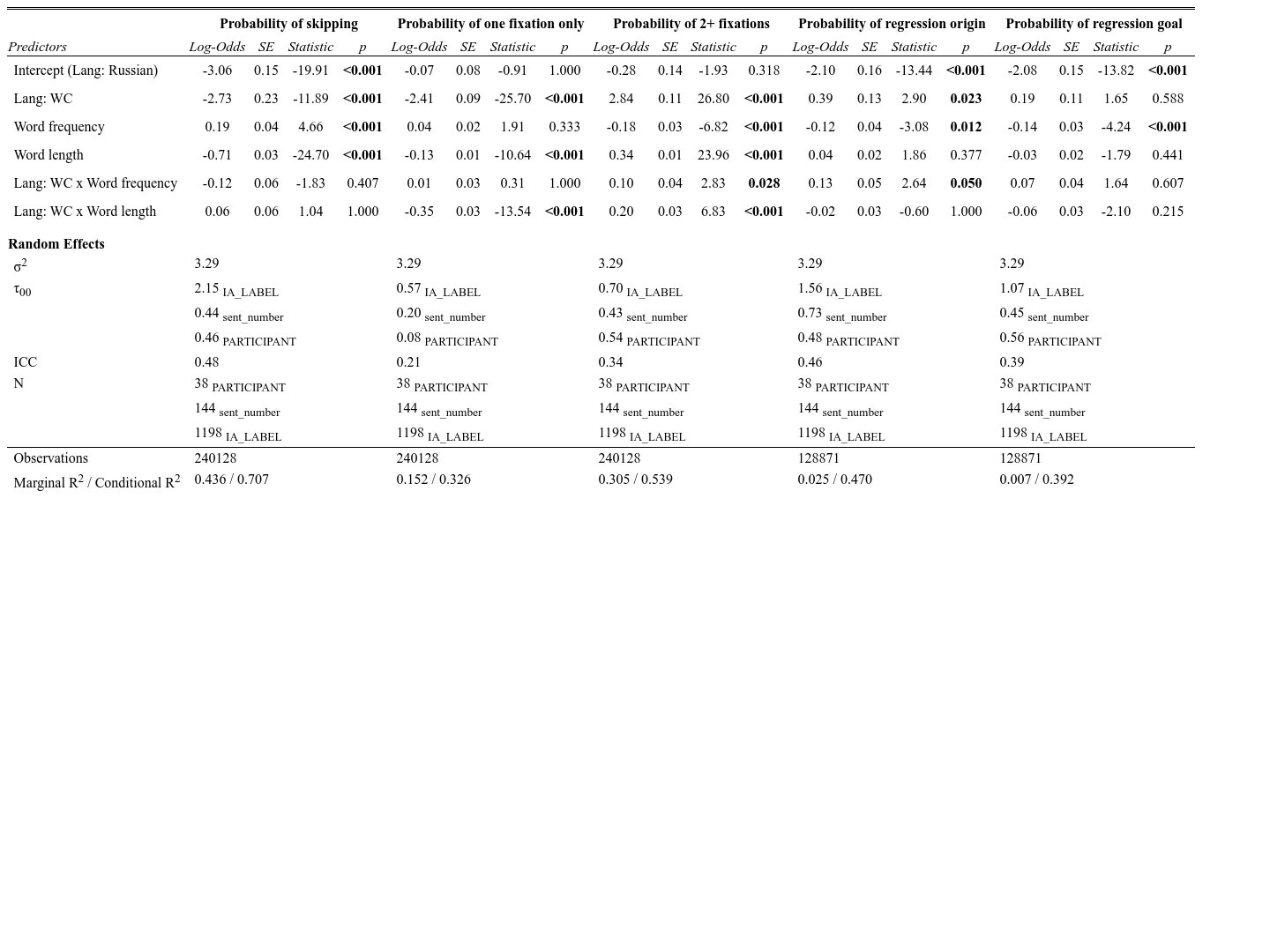

Supplement: Supplementary file 1 [file Data_Sheet_1.docx]
